# Assessing Stigma towards Mental Illness in Relation to Demographics Attitudes and Past Experiences among Pharmacy Students in a Jordanian University Sample

**DOI:** 10.3390/bs13110884

**Published:** 2023-10-25

**Authors:** Amjad H. Bazzari, Firas H. Bazzari

**Affiliations:** 1 Department of Basic Scientific Sciences, Faculty of Arts & Sciences, Applied Science Private University, Amman 11931, Jordan; 2 Faculty of Pharmacy, Jerash University, Jerash 26150, Jordan; f.bazzari@jpu.edu.jo

**Keywords:** stigma, mental health, pharmacy practice, neuropsychiatric disorders, pharmacy education

## Abstract

Stigma towards mental illness poses a significant risk for negative mental health outcomes. Efforts have been undertaken to mitigate self-stigma and stigmatizing behaviors among the public; however, few have considered stigma among healthcare providers, including pharmacists. This study aimed to assess the level of stigma towards mental illness, using the 15-item version of the Opening Minds Scale for Health Care Providers (OMS-HC), and associated factors among pharmacy students and was conducted via a printed questionnaire. A total of 125 students participated and the mean total stigma score was 47.9 with 58.4% of the participants scoring above 45, the midpoint of the possible range of scores. The stigma score was independent of participant demographics, except for grade point average. Higher total stigma scores were observed among subjects who have been prescribed a neuropsychiatric drug before, those who believe that pharmacists should have a role in mental healthcare, those who believe that pharmacists are qualified enough to provide mental health support, and those who are willing to seek help from a pharmacist. The results indicate an overall high stigma score among pharmacy students, which highlights the importance of enhancing pharmacy students’ awareness and knowledge regarding mental healthcare through incorporating additional courses and/or training programs in pharmacy education curricula.

## 1. Introduction

In 2019, it was estimated that one in every eight people worldwide suffer from a mental disorder with depression and anxiety being the most common [1]. According to the Centers for Disease Control and Prevention (CDC), more than one in five adults residing in the United States live with a mental illness and one in 25 suffers from a serious mental disorder, such as schizophrenia, bipolar disorder, and major depression [2]. The prevalence of mental disorders is also found elevated among children and adolescents in Jordan [3]. Furthermore, the coronavirus disease-2019 (COVID-19) had a major impact on the global prevalence of mental health issues, with significantly higher figures compared to reports prior to the pandemic [4]. This continuing increase in prevalence is also coupled with elevations in both economic and disease burdens, which, in turn, calls for the implementation of effective prevention and treatment programs by governments [5,6].

The current status of mental illness, being in the top ten causes of burden worldwide, can be attributed to a long list of challenges facing mental healthcare [5]. Among the major challenges is the stigma of mental illness, which is considered a significant risk factor for negative mental health outcomes [5]. Stigma is found to be responsible for delays in seeking mental support and treatment by individuals and; thereby, can negatively influence therapeutic outcomes [7]. Efforts directed to the public over the years, aiming to spread awareness regarding mental health and reduce stigmatizing behaviors, have achieved positive outcomes in certain populations, especially in the West [8]. Nevertheless, cultural differences among various populations were suggested to take part in the global discrepancies of stigma profiles [9]. Therefore, it has been highlighted that the assessment of stigma is a complex issue with various interactive variables to be considered, including etiological beliefs, attitudes, prejudices, and personal and social problems [10]. Moreover, self-stigma towards the use of neuropsychiatric medications is also observed among patients with mental disorders, which is noted as a leading cause of non-adherence to the treatment and positively correlates with the number of relapses [11,12,13].

On the other hand, self-stigma [14,15,16] and stigmatizing behaviors towards patients with mental illnesses [17,18] have also been observed among healthcare providers. The main sources of stigma in healthcare were previously highlighted in the work of Knaak et al. (2017), which included negative attitudes and behaviors, lack of awareness, therapeutic pessimism, lack of skills, and stigma in workplace culture [19]. These factors further aggravate the negative impact of stigma on both the patients and healthcare providers.

A key to fighting against stigma is establishing a collaborative network among different domains of the healthcare system [19]. Pharmacists are considered the most accessible healthcare providers and play an integral role when it comes to improving patient engagement to care programs and medication stewardship [20]. Multiple international bodies, such as the International Pharmaceutical Federation, Royal Pharmaceutical Society, and Pharmaceutical Society of Australia among others, have established a number of frameworks and highlighted the potential roles of pharmacists in the early detection of mental illness, aiding access to mental health services and optimizing therapies [21]. Nevertheless, pharmacists, as other healthcare providers, are not immune to stigma, and multiple studies have highlighted the importance of identifying and removing barriers in addition to the implementation of solutions to eliminate stigma in pharmacy practice [22,23]. Among the suggested strategies to promote pharmacists’ role in mental healthcare is the early immersion of pharmacy students through training programs in mental healthcare in order to reduce stigma and social distancing and improve attitudes regarding mental illness [24].

Based on the aforementioned findings, and in order to obtain an early depiction of the current status in Jordan, this study aims to assess stigma towards mental illness among pharmacy students in a Jordanian university sample.

## 2. Materials and Methods

### 2.1. Sampling and Ethical Considerations

This cross-sectional study was conducted via a printed questionnaire targeting pharmacy students at Jerash University, Jordan and a total of 125 students participated from a total of around 230 eligible students. The eligibility (i.e., inclusion) criteria were; adult age (18 years or above), active enrollment status in the pharmacy program at Jerash University, and completion of pharmacology (1) course, which covers the various classes of centrally acting agents including neuropsychiatric drugs and mental illnesses.

The survey questionnaire was written in English, the official teaching language for pharmacy programs in Jordan, and was distributed by the researchers among the students during break hours. The participants were given an explanation of the study’s aims and significance prior to handing out the questionnaire and were informed that no personal identifying information would be asked, and the collected data would be solely used for scientific research purposes. Participation was voluntary, and the participants were not paid or compensated and had the right to withdraw at any time. Informed consent was obtained from all subjects involved in the study.

The study was approved by the Department of Pharmacy Council at Jerash University (approval number: 1/ث/2023, date: 6 March 2023), and was conducted with strict adherence to the guidelines of the Declaration of Helsinki regarding anonymity, voluntary participation, and data protection [25].

### 2.2. Questionnaire and Research Instrument

#### 2.2.1. Demographics

The first section of the questionnaire involved the collection of participant demographic variables to investigate their influence on the responses to subsequent sections. These include age in years, gender, self-reported cumulative grade point average (GPA), marital status, smoking status, working in addition to studying status, nationality, and place of permanent residence.

#### 2.2.2. Experiences and Attitudes

The second section aimed to assess the experiences of participants regarding neuropsychiatric disorders and medications. In addition, it also aimed to assess their attitudes and perspectives regarding the current status of the profession in mental healthcare support and their future role as pharmacists, in order to identify areas for improvement in pharmacy education. This section included a total of 7 questions: (1) “Have you known or dealt with someone who has a mental illness?”; (2) “Have you known or dealt with someone who is prescribed a neuropsychiatric drug(s)?”; (3) “Have you ever sought mental health aid by a medical professional?”; (4) “Have you ever been prescribed a neuropsychiatric drug by a medical professional for a specific indication?”; (5) “Do you believe that pharmacists should have a role in providing mental healthcare support to patients?”; (6) “Do you believe that pharmacists are qualified enough to provide mental healthcare support to patients?”; and (7) “If you ever had a mental health disorder, would you seek help from a pharmacist”. All questions had “Yes” and “No” as the potential answer options.

#### 2.2.3. Stigma towards Mental Illness

The 15-item version of the Opening Minds Scale for Health Care Providers (OMS-HC) [26] was utilized to assess the level of stigma towards mental illness among the participants. This questionnaire includes 15 questions scored based on a 5-point Likert agreement scale: “Strongly Agree” scoring 5; “Agree” scoring 4; “Neither Agree nor Disagree” scoring 3; “Disagree” scoring 2; and “Strongly Disagree” scoring 1. However, 5 questions require reverse scoring, which in the original 20-item version of the OMS-HC questionnaire [27] were labeled as items: 3, 8, 9, 10, and 19. Accordingly, each question will receive a score from 1 to 5 and thus the possible range of total stigma scores is from 15 (least stigmatizing) to 75 (most stigmatizing). The questionnaire exhibits a 3-factor structure representing three stigma subscales or domains. These include the attitudes of health care providers towards people with mental illness domain with 6 questions (score range: 6–30); disclosure/help-seeking domain with 4 questions (score range: 4–20); and social distance domain with 5 questions (score range: 5–25). The questions of, and participant responses to, the 15-item OMS-HC questionnaire are described in the corresponding results section. Permission to use the 15-item version of the OMS-HC [26] was obtained via email from Prof. Dr. Andrew Szeto.

### 2.3. Statistical Analysis

Data analysis was conducted using JASP software (Version 0.16.2, www.jasp-stats.org). All results are presented as mean ± standard deviation (SD) or as counts (*n*) and percentages (%). The dependence between participant demographics and gender was assessed using Chi-square (χ^2^) test, except for continuous variables (age and GPA). The normality of distribution was assessed using the Shapiro–Wilk test with a significant result for age (*p* < 0.01) indicating deviation from normality. The participant GPA means were compared using *t*-test while their age ranks were compared using Mann–Whitney U test. The effect size for comparisons of means and ranks (ranks across or grouped by dichotomous variables) were assessed using Cohen’s d and rank-biserial correlation (r_rb_), respectively. The participant responses to the experiences and attitudes questions were coded into ordinal variables and their association with demographic variables was assessed. This was done using Chi-square test except for age and GPA, which were assessed using Spearman’s rank correlation test (rho or ρ statistic). The internal reliability of the 15-item OMS-HC was assessed by calculating Cronbach’s α value. The scores for each question were calculated using the mean of response scores across all participants and the scores for each participant were calculated using the sum of response scores across all or selected questions to obtain total scores and sub-scores (domain scores), respectively. The domain sub-scores of the 15-item OMS-HC questionnaire were compared, after being scaled out of 5, through one-way analysis of variance by ranks using Kruskal-Wallis (KW) test with post-hoc Dunn’s test using Bonferroni-corrected alpha. The non-parametric Mann–Whitney U test and Spearman’s rank correlation test were used to assess the dependence (association and correlation) of total scores on participant experiences and attitudes responses as well as their demographics. All required corrections for the potential influence of confounding variables were done and are described for corresponding tests in the results section. Lastly, all comparison and correlation tests were two-tailed at α error of 0.05 and thus significance was determined at *p* < 0.05.

## 3. Results

### 3.1. Participant Demographics

A total of 125 undergraduate pharmacy students participated with a comparable distribution of males (*n* = 56, 44.8%) and females (*n* = 69, 55.2%). All participant questionnaire responses were complete and thus none were omitted. The results are presented as mean ± standard deviation (SD) or as counts and percentages (%). The mean age of participants was 22.52 years (SD ± 2.69), ranging from 19 to 35 years. The age ranks did not differ between male (mean: 22.75 ± 2.87) and female participants (22.33 ± 2.55, *p* > 0.05, d = 0.16, r_rb_ = 0.104). Many other demographic factors were collected to assess their potential association with participant experiences and attitudes regarding mental disorders and their potential influence on participants’ stigma towards mental illness. The cumulative GPA of the participants ranged from 57.9 to 92.7% and the mean was higher for females (76.28 ± 6.79%) compared to males (72.93 ± 7.32%, *p* < 0.01, d = 0.48). The majority of participants are Jordanian (55.2%), permanently reside in Jordan (66.4%), and reported being single (91.2%), all of which is true for males and females; thus, independent from gender (*p* > 0.05). However, male participants had a significantly higher distribution of smoking (46.4%, *p* < 0.01) and working students (60.7%, *p* < 0.01) compared to females (10.1% and 31.9%, respectively). All collected demographic variables and participant responses are summarized in Table 1.

### 3.2. Personal Experiences and Attitudes

Following the collection of demographics, the participants were prompted to answer seven questions that aimed to assess their personal experiences in regard to neuropsychiatric disorders and medications in addition to their attitudes toward the role of pharmacists as healthcare professionals in mental health support. Most participants report having known or dealt with someone with a mental illness (68.8%) or administering a prescribed neuropsychiatric drug (59.2%) previously. In both cases, a positive response was significantly higher for males (78.6% and 76.8%) compared to female participants (60.9% and 44.9%, respectively, *p* < 0.05). A similar association was observed for working status as more working students had a previous interaction (78.6% and 73.2%) than non-working students (60.9% and 47.8%, respectively, *p* < 0.05). This association explains the impact of gender, which was lost when corrected for working status (*p* > 0.05). When the participants were asked whether they have ever sought mental health aid most responses were negative (70.4%), which did not significantly differ between males (62.5%) and females (76.8%, *p* > 0.05) or across any other demographic factor. On the other hand, a significantly higher number of males report that they have been prescribed a neuropsychiatric drug before (30.4%) compared to females (13%, *p* < 0.05). Interestingly, having been prescribed a neuropsychiatric drug was negatively correlated, to a weak but statistically significant degree, with GPA (ρ = −0.293, *p* < 0.001) and the correlation remained significant despite correction for gender (ρ = −0.241, *p* < 0.01). Indeed, the mean and rank distribution of the GPA of students who have not been prescribed a neuropsychiatric drug (75.8 ± 7.44%) was higher than those who have (70.9 ± 4.48%, *p* < 0.01, d = 0.71, r_rb_ = 0.423). Aside from gender and GPA, the responses to this question were independent of all other demographic variables. The last part of the experiences and attitudes section focused on participant perceptions towards pharmacists in relation to mental healthcare support. The vast majority of participants (91.2%) do believe that pharmacists should have a role in providing mental healthcare support to patients, which was independent of, and therefore true across all participant demographics except for age, which to a weak degree was negatively correlated with the support for a pharmacist role (ρ = −0.221, *p* < 0.05, d = 0.81, r_rb_ = 0.443). A lesser majority of participants (63.2%); however, believe that pharmacists are qualified enough to provide mental healthcare support to patients, which varied based on gender, GPA, marital status, and place of permanent residence. The belief of adequate pharmacist qualification was negatively correlated with GPA (ρ = −0.272, *p* < 0.01, d = 0.58, r_rb_ = 0.326) and was higher among males (76.8%, *p* < 0.01), single participants (66.7%, *p* < 0.05) and students whose permanent residence is not in Jordan (76.2%, *p* < 0.05) compared to females (52.2%), married participants (27.3%) and students whose permanent residence is in Jordan (56.6%). Lastly, a comparable distribution, independent from all collected participant demographic variables (*p* > 0.05), was observed between participants who would (52.8%) and would not (47.2%) seek help from a pharmacist if they ever had a mental health disorder. The participant responses to personal experiences and attitudes questions and the impact of demographics, except for smoking status as it did not have any form of impact on participant responses, are summarized in Table 2.

### 3.3. Stigma towards Mental Illness: OMS-HC Score

The stigma towards mental illness among the participants was assessed using the 15-item OMS-HC questionnaire, which showed adequate internal reliability (α = 0.724), and total scores were calculated for each participant. The mean total stigma score of all participants was 47.9 ± 8.49 (or 3.19 ± 0.566 out of 5), ranging from 21 to 75 with 73 participants (58.4%) scoring above 45, which is the midpoint of the possible range of scores (from 15 to 75). The sub-scores of the three domains of the 15-item OMS-HC questionnaire were also calculated and averaged (out of 5) for each and across all participants. The highest mean score of 17.16 ± 3.43 (or 3.43 ± 0.69 out of 5) was observed for the social distance domain followed by the disclosure/help-seeking domain with a mean score of 12.5 ± 2.92 (or 3.12 ± 0.73 out of 5) and lastly the attitudes of health care providers towards people with mental illness domain with a mean score of 18.26 ± 4.33 (or 3.04 ± 0.72 out of 5). Analysis of variance by ranks across the three domains was significant (*p* < 0.01) and post-hoc analysis shows that the social distance ranks are significantly higher than the attitudes and disclosure domains (*p* < 0.01). A summary of response scores for the 15-item OMS-HC questionnaire is provided in Table 3.

**Figure 1:**
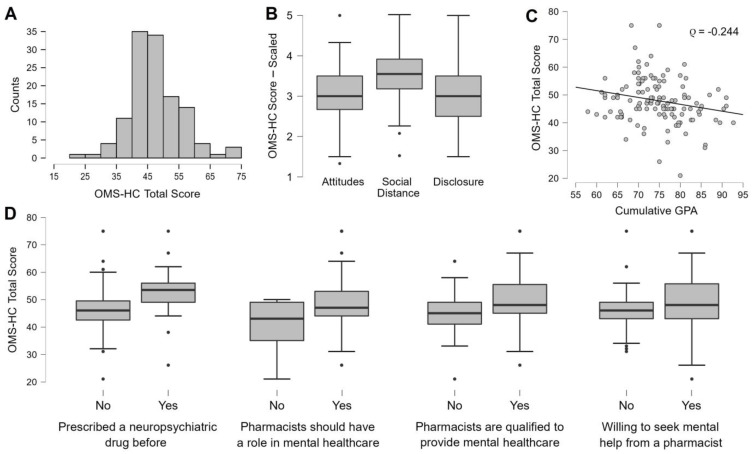
Summary plots of OMS-HC score results. The figure shows (**A**) the distribution of total OMS-HC scores, (**B**) boxplots of OMS-HC domain sub-scores scaled out of 5 (the dots represent the outliers), (**C**) correlation plot between total OMS-HC scores and cumulative GPA (the dots represent the data points) and (**D**) boxplots of the personal experiences and attitudes that significantly affected total score ranks.

In relation to participant demographics, the total stigma score varied by rank, only based on cumulative GPA and thus was independent of gender, age, marital status, working status, smoking status, nationality, and place of permanent residence of the participants. Regarding the cumulative GPA, it was found to negatively correlate, but to a low degree, with a total stigma score (ρ = −0.244, *p* < 0.01); additionally, the rank correlation remained statistically significant when conditioned on gender (*p* < 0.05) considering the variation of GPA between male and female participants. In relation to participant experiences and attitudes, four main factors had a statistically significant effect on participants’ stigma towards mental illness favoring higher total stigma score ranks. These were: having been prescribed a neuropsychiatric drug before (mean: 52.89 ± 9.03 compared to 46.62 ± 7.88, *p* < 0.01, d = 0.771, r_rb_ = 0.52), believing that pharmacists should have a role in mental healthcare support (48.6 ± 8.13 compared to 40.91 ± 9.3, *p* < 0.01, d = 0.934, r_rb_ = 0.4), believing that pharmacists are qualified enough to provide mental healthcare support (49.67 ± 8.74 compared to 44.91 ± 7.17, *p* < 0.01, d = 0.58, r_rb_ = 0.334) and willingness to seek help from a pharmacist in case of having a mental health disorder (49.32 ± 9.29 compared to 46.36 ± 7.25, *p* < 0.05, d = 0.353, r_rb_ = 0.206). The OMS-HC score results and associated demographic and personal attitudes and experiences factors are summarized in Figure 1.

## 4. Discussion

This study is the first to explore stigma towards mental illness and associated demographics, attitudes, and past experiences among pharmacy students in Jordan. The results revealed an overall elevated level of stigma towards neuropsychiatric disorders, with 58.4% of the sample participants exhibiting elevated stigma scores. Among the assessed factors, four were found to be associated with high stigma scores; including (1) having been prescribed a neuropsychiatric drug before; (2) believing that pharmacists should have a role in mental healthcare; (3) believing that pharmacists are qualified enough to provide mental health support; and (4) willingness to seek help from a pharmacist for a mental health disorder. This, in turn, indicates an association of participants’ stigma towards mental illness with perceptions towards the qualification of and confidence in pharmacists. However, except for GPA, none of the collected demographic factors had any significant influence on stigma scores.

The majority of the participants believed that pharmacists should have a role in mental healthcare support and that pharmacists are qualified enough to provide mental support services with approximately half of the sample stating that they would seek help from a pharmacist in this regard. These findings further support the awareness of pharmacists and the positive attitude toward their key roles in mental healthcare and the beneficial impact on therapeutic outcomes [28]. These include recognizing mental illness and providing resources and referrals for the patients in need, as well as their roles in enhancing medication adherence and medication review [29].

The mean total stigma score obtained via the OMS-HC was 47.9, in which the social distance domain had the highest mean score followed by the disclosure and attitudes domains. Nonetheless, the stigma score was independent from all demographics except for GPA, which had a negative correlation. When compared to other populations in previous studies that adopted the 15-item OMS-HC, the results indicate a relatively high stigmatizing behavior among the study subjects. For instance, in the work of Douglass and Moy (2019) evaluating the impact of a social media-focused intervention on mental health stigma among a university sample of pharmacy students in the United States, both pre- and post-intervention scores (36.8 and 35.1, respectively) are markedly lower compared to the current results [30]. Among working pharmacists in Accra, the overall score was 37, which is also significantly lower in comparison to the current findings [31]. This can also be observed among other populations from different domains, such as medical students in Saudi Arabia [32], nursing students in Australia [33], and other primary healthcare providers in Bahrain and Chile [34,35]. In all of the aforementioned studies that used the OMS-HC, and consistent with the findings herein, there was no statistically significant influence of gender on the stigma scores of the participants. However, in other studies that used different stigma assessment tools, the results differed in terms of gender impact on stigma towards mental illness, as some studies reported the existence of a gender impact [36,37], while others reported no influence [38,39]. The assessment and/or inclusion of other demographics (i.e., nationality, marital status, etc.) varied considerably in the literature [40,41,42,43,44], and, therefore, this calls for further large-scale studies to assess their potential influence.

Interestingly, higher total stigma scores were observed among subjects who have been prescribed a neuropsychiatric drug before, believe that pharmacists should have a role in mental health support, believe that pharmacists are well qualified to provide mental health support, and are willing to seek help from a pharmacist. While no definitive explanations for these findings can be drawn at this point, some evidence may provide further insights into the matter. For instance, the belief that pharmacists are well qualified to provide mental health support and the willingness of participants to seek help from a pharmacist may imply a case of high self-esteem, which is found to be associated with increased stigmatization [45]. Having been prescribed a neuropsychiatric drug and elevated stigmatizing behavior may seem paradoxical; on one hand, it could be initially assumed that a past experience may increase empathy [46] and, in turn, lower the level of stigma [47]: however, this was not the case herein. The work of Ruttan et al. (2015) elaborated further on such situations, highlighting that “having been there doesn’t mean I care”; in other words, individuals who had previously experienced and endured an emotionally distressing event tend to harshly evaluate others’ failure to endure a similar event [48], and in this case, it may, perhaps, be translated into an elevated stigmatizing behavior. From another perspective, this may be in relation to self-stigma, as it was previously argued that self-stigma poses a paradox in reactions towards one’s expression, as some may lose self-esteem while others become “energized by prejudice and express righteous anger” [49]. Nevertheless, future investigations are warranted to provide further insights into these findings.

A relationship between stigmatizing behaviors and decreased willingness to provide effective clinical services to patients was noted among pharmacists previously; nevertheless, increased interaction and familiarity combined with further education and literacy were observed to have a de-stigmatizing effect [50,51,52]. From a national point of view, and consistent with the current results, the work of Abdel-Qader et al. (2021) among Jordanian pharmacists has revealed an overall positive attitude towards providing pharmaceutical care to patients with psychiatric disorders; however, there was a general lack of confidence and proper knowledge in terms of psycho-pharmacotherapy [53]. Therefore, a consensus can be observed among the literature regarding the importance of incorporating further education at all levels (both under- and post-graduate) and increased familiarity with patients suffering from mental illnesses in reducing stigma and improving provided pharmaceutical services.

The current study has a number of limitations: (1) the sample was based in one university and several other institutions with BSc. pharmacy programs should be included as well in future studies, (2) the sample did not include Doctor of Pharmacy (PharmD.) students in Jordan who can fulfill similar roles, and (3) several socio-demographic factors, such as economic status and cultural and social beliefs *etc.*, which may influence participant responses and attitudes, were not collected and may aid future investigations. However, this work provides an initial depiction regarding the attitudes and level of stigma towards mental illness among pharmacy students in Jordan, which calls for the implementation of various strategies to mitigate stigma towards mental illness, and this shall include interventions at different levels ranging from improvements in pharmacy education to the adoption of anti-stigma health policies and/or national frameworks to govern the clinical practice.

## 5. Conclusions

In conclusion, the results of the current study indicate an overall high stigma score among pharmacy students, which is independent of demographic factors, except for GPA. In relation to past experiences and attitudes towards the role of pharmacists in mental healthcare, higher stigma scores are observed among subjects who have been prescribed a neuropsychiatric drug before, believe that pharmacists should have a role in mental health support, believe that pharmacists are well qualified to provide mental health support and are willing to seek help from a pharmacist. However, positive attitudes are observed among the vast majority with regard to the further incorporation of pharmacists and the expansion of their roles in mental healthcare. These findings, thereby, highlight the importance of enhancing pharmacy students’ awareness, knowledge, and familiarity regarding the various aspects of mental healthcare through incorporating additional courses and/or training programs in undergraduate pharmacy education curricula.

## Figures and Tables

**Table 1:** Participant Demographics.

Variable	Total	Males	Females	*p* Value
Age in years: mean (SD)	22.52 (2.69)	22.75 (2.87)	22.33 (2.55)	0.31 ^a^
GPA: mean (SD)	74.78 (7.2)	72.93 (7.32)	76.28 (6.79)	0.009 ^b,^*
Marital Status: count (%)				0.221 ^c^
Single	114 (91.2%)	53 (94.6%)	61 (88.4%)	
Married	11 (8.8%)	3 (5.4%)	8 (11.6%)	
Smoking: count (%)				<0.001 ^c,^*
No	92 (73.6%)	30 (53.6%)	62 (89.9%)	
Yes	33 (26.4%)	26 (46.4%)	7 (10.1%)	
Working: count (%)				
No	69 (55.2%)	22 (39.3%)	47 (68.1%)	0.001 ^c,^*
Yes	56 (44.8%)	34 (60.7%)	22 (31.9%)	
Nationality: count (%)				0.742 ^c^
Jordanian	69 (55.2%)	30 (53.6%)	39 (56.5%)	
International	56 (44.8%)	26 (46.4%)	30 (43.5%)	
Residence: count (%)				0.406 ^c^
Jordan	83 (66.4%)	35 (62.5%)	48 (69.6%)	
Other	42 (33.6%)	21 (37.5%)	21 (30.4%)	

^a^ Mann–Whitney test, ^b^ *t*-test, ^c^ Chi-square test, * Significant (*p* < 0.05).

**Table 2:** Impact of participant demographics on their experiences and attitudes regarding neuropsychiatric disorders, medications, and role of pharmacists.

Questions and Responses		Impact of Participant Demographics ^a^ (*p* Value ^b^)						
		Gender	Age	GPA	Marital Status	Working Status	Nationality	Permanent Residence
Q1. Have you known or dealt with someone who has a mental illness?								
Yes	68.8%	0.034 *	0.048 *	0.122	0.768	0.034 *	0.855	0.39
No	31.2%							
Q2. Have you known or dealt with someone who is prescribed a neuropsychiatric drug(s)?								
Yes	59.2%	<0.001 *	0.275	<0.001 *	0.339	0.004 *	0.297	0.048 *
No	40.8%							
Q3. Have you ever sought mental health aid by a medical professional?								
Yes	29.6%	0.081	0.615	0.361	0.385	0.575	0.82	0.515
No	70.4%							
Q4. Have you ever been prescribed a neuropsychiatric drug by a medical professional for a specific indication?								
Yes	20.8%	0.018 *	0.761	<0.001 *	0.316	0.876	0.876	0.555
No	79.2%							
Q5. Do you believe that pharmacists should have a role in providing mental healthcare support to patients?								
Yes	91.2%	0.556	0.013 *	0.515	0.25	0.188	0.556	0.257
No	8.8%							
Q6. Do you believe that pharmacists are qualified enough to provide mental healthcare support to patients?								
Yes	63.2%	0.005 *	0.121	0.002 *	0.01 *	0.884	0.086	0.032 *
No	36.8%							
Q7. If you ever had a mental health disorder, would you seek help from a pharmacist?								
Yes	52.8%	0.216	0.836	0.947	0.609	0.05	0.355	0.947
No	47.2%							

^a^ Smoking status is not included (insignificant across all questions), ^b^ Chi-square test except for age and GPA (Spearman’s correlation test), * Significant (*p* < 0.05).

**Table 3:** Participants’ response scores for the 15-item OMS-HC questionnaire.

Domains and Questions	Score/5
Factor 1: Attitudes of health care providers towards people with mental illness	3.04
I am more comfortable helping a person who has a physical illness than I am helping a person who has a mental illness	3.71
Despite my professional beliefs, I have negative reactions towards people who have mental illness	2.62
There is little I can do to help people with mental illness	3.06
More than half of people with mental illness don’t try hard enough to get better	3.42
Health care providers do not need to be advocates for people with mental illness	2.73
I struggle to feel compassion for a person with a mental illness	2.72
Factor 2: Disclosure/help-seeking	3.12
If I were under treatment for a mental illness I would not disclose this to any of my colleagues	3.47
I would see myself as weak if I had a mental illness and could not fix it myself	2.94
I would be reluctant to seek help if I had a mental illness	3.05
If I had a mental illness, I would tell my friends	3.04 *
Factor 3: Social Distance	3.43
If a colleague with whom I work told me they had a managed mental illness, I would be as willing to work with him/her	3.78 *
Employers should hire a person with a managed mental illness if he/she is the best person for the job	3.32 *
I would still go to a physician if I knew that the physician had been treated for a mental illness	3.19 *
I would not want a person with a mental illness, even if it were appropriately managed, to work with children	3.42
I would not mind if a person with a mental illness lived next door to me	3.44 *
Total Score	3.19

* Reverse-scored.

## Data Availability

Available on request from the corresponding author, A.H.B.
